# Use of Injectable Carboxymethylcellulose Gel in the Treatment of Acquired Bronchoesophageal Fistula Secondary to Lung Cancer

**DOI:** 10.1016/j.atssr.2025.11.013

**Published:** 2025-12-08

**Authors:** Kaity Han-Yung Tung, Chancy Fontenelle, Max Ilecki, Nathaniel Ivanick, Andrew Bain, Kenneth Patrick Seastedt

**Affiliations:** 1 Department of Surgery, University at Buffalo, Buffalo, New York; 2 Department of Thoracic Surgery, Roswell Park Comprehensive Cancer Center, Buffalo, New York; 3 Department of Internal Medicine–Gastroenterology, Roswell Park Comprehensive Cancer Center, Buffalo, New York

## Abstract

Bronchoesophageal fistula (BEF) is a rare but serious complication of malignant neoplasia of the lung. Whereas endoscopic stenting has emerged as a less invasive alternative, persistent or complicated fistulas may require adjunctive therapies. We present a case of successful closure of a 4-mm BEF secondary to stage IIIB left lung squamous cell carcinoma in a 64-year-old man, refractory to stenting and endoscopic suturing, achieved without surgery. This case highlights the potential role of carboxymethylcellulose gel as a minimally invasive adjunct to stents in management of small yet persistent BEFs.

Acquired bronchoesophageal fistula (BEF) is a rare but life-threatening manifestation of both benign and malignant processes.^1^ The standard treatment involves suture ligation of the fistula with or without pedicled flap coverage and some extent of lung resection by thoracotomy if involvement of lung parenchyma is evident.^2^ However, there has been a shift toward endoscopic management of BEF with bronchial or esophageal stents.^3^ This approach aims to prevent soilage while promoting closure of the fistulous tract through inflammation and granulation. Stenting also serves as a palliative measure, sealing the fistula, alleviating respiratory distress and dysphagia, and preventing recurring infection. Whereas endoscopic stent management has shown promising results, it may still be insufficient in some cases, particularly larger or persistent fistulas or those in challenging locations, necessitating additional interventions such as surgical repair. To address these cases, adjunctive use of clips, plugs, endoscopic suturing, and sealants in addition to stenting has been applied in selective cases of BEF. Here, we present a case of successful closure of smaller though persistent BEF using an injectable carboxymethylcellulose gel, Prolaryn (Merz Therapeutics), as an adjunct, avoiding the need for surgical intervention with high morbidity.

A 64-year-old man with past medical history of tobacco use of 40 pack-years and hypertension was diagnosed with left-sided squamous cell lung cancer after presenting with obstructive pneumonia secondary to complete left mainstem bronchus occlusion as seen in Figure 1A, necessitating bronchial stent placement. Subsequent clinical staging workup revealed stage IIIB disease, and the patient underwent definitive chemoradiation therapy followed by immunotherapy as he was not a surgical candidate. Two months later, chest computed tomography showed extraluminal air adjacent to the stent, concerning for fistula (Figure 1B). Bronchoscopy and esophagogastroduodenoscopy identified a 10-mm BEF distal to the carina in the medial wall of the left mainstem bronchus and 30 cm from the incisors with evidence of reactive inflammation and granulation (Figures 1C and 1D, respectively). Closure with X-Tack (Boston Scientific) suturing and airway stent replacement were initially performed by the gastroenterology and interventional pulmonology care teams (Figure 1E). Despite minimal symptoms, however, a 5-month surveillance computed tomography scan revealed persistent fistula (Figure 1F). Before proceeding with definitive surgical repair, repeated bronchoscopy confirmed a smaller but persistent 4-mm BEF (Figure 1G), and biopsy of the fistulous tract excluded malignant transformation. An in-depth discussion was had with the patient weighing the risks of a minimally invasive approach using adjunctive Prolaryn injection, which is approved by the US Food and Drug Administration for vocal cord insufficiency, against that of esophagectomy. The black box warning pertaining to the injection of carboxymethylcellulose gel into airways was explained to the patient as risks were perceived to be small. The patient expressed understanding and a preference for the minimally invasive approach. Bronchoscopic Prolaryn injection was administered around the fistulous opening with fluoroscopic confirmation (Figure 1H), and the stent was replaced. Two months later, endoscopy confirmed complete healing of the BEF, and the stent was removed without complication (Figure 1I). The patient remains clinically stable and continues immunotherapy, expressing satisfaction with having avoided invasive surgery. The timeline of the clinical course is summarized and depicted in Figure 2.

**Figure 1:**
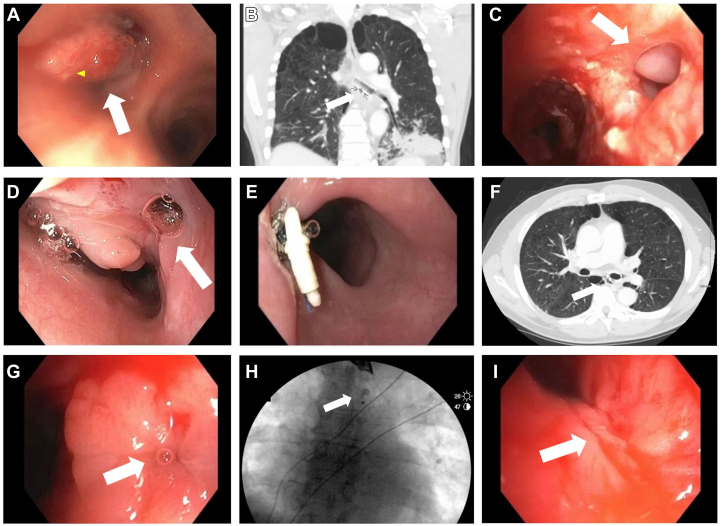
(A) Pretreatment rigid bronchoscopy was used to diagnose the original left mainstem bronchus tumor indicated by both yellow and white arrows. (B) A screenshot of a coronal section of the chest computed tomography scan showed extraluminal air (arrow) adjacent to left mainstem bronchial stent. (C) Bronchoscopy showed the 10-mm bronchoesophageal fistula (BEF; arrow) in the medial wall of the left mainstem bronchus just distal to the carinal bifurcation. (D) Esophagogastroduodenoscopy also showed the 10-mm BEF (arrow) 30 cm from the incisors. (E) X-Tack suturing was used for the initial closure of the fistula. (F) A screenshot of an axial section of the 5-month surveillance chest computed tomography scan showed the persistent fistulous tract (arrow). (G) Repeated bronchoscopy showed the persistent 4-mm fistula opening (arrow). (H) Prolaryn gel was injected into the fistula and confirmed on fluoroscopy (arrow). (I) Repeated bronchoscopy 2 months after showed completely healed BEF (arrow).

**Figure 2:**
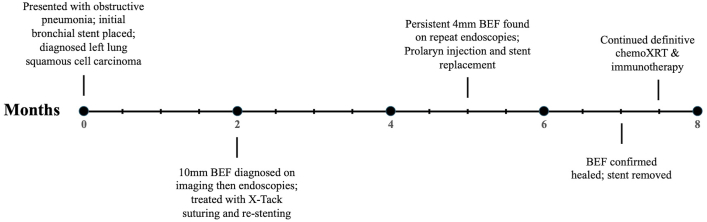
Timeline depiction of clinical course of events. (BEF, bronchoesophageal fistula; chemoXRT, chemoradiation therapy.)

## Comment

Acquired BEF is an uncommon but serious presentation secondary to esophageal and pulmonary malignant neoplasms. The aligned goal of treatment is prompt closure of the fistulous tract to prevent risks of chronic infections and progressive airway or esophageal destructions. However, it remains a challenging pathologic process as patients are typically in a chronic malnourished state with impaired healing capacity, conditions frequently observed in the setting of malignant disease and concurrent chemoimmunotherapy. Although there has been a paradigm shift toward endoscopic management of BEFs, stent placement alone is not universally effective and may be associated with complications or incomplete healing. In such cases, adjunctive techniques may be required to further stimulate granulation tissue formation and inflammation for durable closure. Minimally invasive management should be considered and trialed early if appropriate before committing patients to operations associated with high morbidity.

Several novel adjuncts have been reported in the literature. Ariza-Prota and coworkers^4^ used an Amplatzer device (Abbott Cardiovascular) as a bridging therapy to combat a 2.5-cm BEF in the medial wall of the intermediate bronchus that arose secondary to esophageal diverticulum, allowing eventual definitive surgical repair once the patient’s condition was optimized. Smesseim and coworkers^5^ reported the novel use of a 3-dimensional printed airway stent in addition to an esophageal stent after failure of conventional stenting. Akkawi and coworkers^6^ described successful use of Prolaryn gel in addition to DuraSeal glue (Integra), postpyloric feeding, and hyperbaric oxygen therapy to treat a persistent 2-mm BEF after failure of closure with stent only management. These cases highlight that the choice of adjunctive therapy should be tailored to fistula size, location, and patient condition.

Our case demonstrates successful closure of a persistent 4-mm BEF with Prolaryn injection after prior unsuccessful treatment of bronchial stenting and X-Tack endoscopic suturing. Administration of Prolaryn injection at the fistula site alone in our case resulted in complete healing, supporting its utility as a minimally invasive adjunct or appropriate substitute to esophageal stenting. However, this is under the pretense that malignant transformation has been ruled out from the fistula. The generalizability and applicability threshold of this adjunct to type of BEF have yet to be determined. From our experience, the injection of Prolaryn gel was precisely directed to the fistula site under both bronchoscopic and fluoroscopic guidance with the assistance of sclerotherapy or endoscopic ultrasound needle. Moreover, the gel was administered in small aliquots of approximately 0.25 mL until complete closure of the defect was observed. Incremental administration under direct visualization reduced the risk of extravasation or leakage of the Prolaryn gel into the airway, preventing unwanted obstruction or mucosal bulging.

In conclusion, the management of BEF secondary to lung cancer requires a multidisciplinary and multimodality approach. In cases of small but persistent fistulas, carboxymethylcellulose injection may serve as a minimally invasive adjunct to endoscopic stenting before consideration of extensive surgery.
